# The urgency for a change in genetics healthcare provision: views from Portuguese medical geneticists

**DOI:** 10.1007/s12687-024-00702-1

**Published:** 2024-03-01

**Authors:** Catarina Costa, Lídia Guimarães, Ruxanda Lungu Baião, Marina Serra de Lemos, Luís Filipe Azevedo, Milena Paneque

**Affiliations:** 1 i3S-Institute for Research and Innovation in Health, University of Porto, R. Júlio Amaral de Carvalho, 45, Porto, 4200-135 Portugal; 2 IBMC-Institute of Molecular and Cellular Biology, University of Porto, Porto, Portugal; 3 CGPP-Center for Predictive and Preventive Genetics, University of Porto, Porto, Portugal; 4 FMUP-Faculty of Medicine, University of Porto, Porto, Portugal; 5 ICBAS-School of Medicine and Biomedical Sciences, University of Porto, Porto, Portugal; 6 AAJUDE – Associação de Apoio à Juventude Deficiente, Porto, Portugal; 7 FPCEUP-Faculty of Psychology and Educational Sciences, University of Porto, Porto, Portugal; 8 CPUP-Center for Psychology, University of Porto, Porto, Portugal; 9 MEDCIDS-Department of Community Medicine, Health Information and Decision Sciences, Faculty of Medicine, University of Porto, Porto, Portugal; 10 CINTESIS@RISE-Center for Health Technology and Services Research, University of Porto, Porto, Portugal

**Keywords:** Enetics services, Genomics, Health policy, Health services accessibility, Human genetics, Public health

## Abstract

In the last decades, genetics has experienced significant technological advancements worldwide. However, in Portugal, serious limitations persist, compromising the functioning of healthcare in medical genetics. This study aimed to promote sharing and discussion among genetic medical professionals, to outline concrete actions to address gaps in clinical practice. Three focus groups were conducted with 19 specialists in medical genetics. The data were analyzed using the thematic analysis method to extract the main themes from the discussions. From the analysis, four conceptual themes emerged: (i) framing Portuguese genetic services in light of the European context; (ii) improvement of medical genetics education and population literacy; (iii) transforming of medical genetics services; and (iv) operationalizing the change. The results demonstrated that increasing training resources and strengthening multiprofessional teams by hiring more genetic professionals, such as clinical geneticists, molecular geneticists, and other genetic specialists, is crucial to enhancing the responsiveness of genetic services. Integrating medical genetics into all specialties and primary care, as well as updating the national network of medical genetics, are critical points for increasing equity and enabling healthcare to be provided more fairly. Including other medical genetics professionals such as genetic counsellors, nurses and psychologists also plays a significant role in providing comprehensive and quality care. This collaborative approach aims to provide effective genetic assistance and enhance the adequacy of genetic healthcare. The findings are compiled as recommendations to support the profession moving forward that can be applied to other healthcare contexts worldwide.

## Introduction

Medical genetics has evolved exponentially in the last twenty years, leading to major scientific breakthroughs and advancements (Horton and Lucassen 2019). Several European Rare Disease Reference Networks have been created in the field, and humanity has witnessed the astonishing impact of genomics’ emergence on healthcare systems worldwide (Horton and Lucassen 2019). However, a long path is yet to be covered, especially regarding technical aspects and service structures.

Indeed, while the rarity of some diseases poses challenges regarding diagnosis and necessary research for developing appropriate treatments (Diário da República 2015), directing patients to specialized genetic services remains an inefficient process in Portugal. This lack of referral capacity might be related to the absence of a solid genetic counselling structure supported by the creation of reference centers (Diário da República 2015).

The increasing demand for genetic consultations is coupled with severe limitations of the few existing genetic services in Portugal (Costa et al. 2023). In Portugal, the healthcare system, represented by five Regional Health Administrations (ARS) units – North, Centre, Lisbon and Tagus Valley, Alentejo, and Algarve – acts as an intermediary between the Ministry of Health and local structures. Public funding, under Law No. 95/2019 dated September 4th, integrates genetic services into the National Health Service (SNS), covering salaries, consultations, and genetic tests.

Most genetics services are in reference hospitals in the country’s main cities, such as Lisbon, Porto, and Coimbra. National genetic services have historically been integrated into tertiary care, requiring referrals to central services, which translates into various barriers to access – geographical, financial, and psychological (Hawkins and Hayden 2011). Healthcare professionals such as pediatricians and oncologists identifying the need for genetic evaluation refer their patients to genetic services. Although genetic tests conducted in public services are funded by the SNS, the associated waiting times for consultations and results are typically longer than in private services, which also offer medical genetic consultations.

National public laboratories, such as the Dr. Ricardo Jorge National Institute of Health (INSA), conduct genetic tests, and when specific tests are unavailable locally, they may be outsourced abroad. The preliminary interpretation of test results falls under the purview of clinical laboratory geneticists, with the final communication to patients always conducted by medical geneticists.

The number of medical genetics professionals in the services falls short of the needs, with only 48 specialists serving the entire country. Considering the international criterion of the Royal College of Physicians of the United Kingdom, which suggests a ratio of 6 to 12 genetic professionals per million inhabitants (Abacan et al. 2018), between 66 and 132 medical geneticists would be required. On the other hand, genetic counsellors and genetic nurses are not officially recognized as professionals by national authorities and although there is a MSc program for training such professionals they are not all integrated in genetics healthcare (Costa et al. 2023).

Theoretically, one of the best ways to incorporate genetics more efficiently and equitably into healthcare is by creating structures that allow patients to access genetic services within the context of primary care (Chou et al. 2021). However, in European countries like Portugal general practitioners’ technical and scientific skills must first be created for these structures to be of actual value (Carroll et al. 2019).

With a growing imbalance between supply and demand, alternative models of genetic service provision are needed. Thus, over the past years, multidisciplinary work has been developed to evolve the efficiency and quality of medical genetics and genetic counselling, seeking to identify and suggest changes to structural and human shortcomings (Paneque et al. 2018, 2021). The most recent study in Portugal, conducted with national directors of genetics services, demonstrates that medical genetics healthcare needs to redefine its place in the healthcare system and improve the organization and composition of services, based on a multidisciplinary, multiprofessional and patient-centered approach (Costa et al. 2023).

Therefore, considering the entire panorama presented previously, we designed this study to contribute to the definition of a new model for medical genetics service delivery in Portugal, reflecting upon (1) the need for healthcare change, (2) the influence of the European context, (3) the critical entities in the reorganization of medical genetics, (4) the necessary strategies, and (5) the concrete actions to enhance the change.

## Materials and methods

We chose a qualitative methodology for this exploratory, descriptive study by conducting three online focus groups. The qualitative approach offers more comprehensive perspective of the subject, particularly relevant when generating and delving into content related to a specific research line.

One of the significant advantages of this method lies in the group dynamics adopted, allowing participants not only to express their own ideas, but also to develop them in interaction with others. By sharing experiences and engaging in differentiated discussions, participants were able to deepen their reflection on their understanding of the topic in question (Stewart and Shamdasani 2014).

### Participants

Convenience sampling was employed to recruit medical geneticists from various genetic services in Portugal. All directors of public medical genetics services and medical geneticists meeting any of the following inclusion criteria were invited to participate:

1. Specialist in medical genetics practicing within a genetics service in Portugal;
2. Member of Specialty College of Medical Genetics board;
3. Member of Portuguese Society of Human Genetics board.

The sole exclusion criterion was:

(1) Medical intern currently undergoing postgraduate training, acquiring practical experience (5 years) under supervision in the field of medical genetics. These professionals were excluded as they do not possess yet a comprehensive view of the national context in genetics healthcare.

Overall, nineteen medical geneticists participated in this study, corresponding to 39% of medical geneticists in Portugal. Three of them are specialists working as service directors and one is the director of one genetic clinics in the process of obtaining service status.

These professionals practiced medical genetics in the following Portuguese genetic services: University Hospital Center of Coimbra (Centro Hospitalar Universitário de Coimbra), University Hospital Center of São João (Centro Hospitalar Universitário de São João), University Hospital Center of Central Lisbon (Centro Hospitalar Universitário de Lisboa Central), University Hospital Center of North Lisbon (Centro Hospitalar Universitário Lisboa Norte), Hospital of Braga and Portuguese Institute of Oncology – Porto (IPO- Porto).

### Study Design and Data Collection

First, an email was sent to all directors of genetics services across the country, inviting their specialists to participate and explaining the study’s objectives. They were asked to indicate their availability for engaging in an online focus group and to identify additional team members who met the criteria for participation.

We conducted three online semi-structured focus groups with pre-selected themes. Questions were asked about the current context of genetics in Portugal, specifically (a) the need for a change in healthcare, (b) the European context, (c) key entities in the reorganization of the field, (d) necessary strategies, and (e) concrete actions for change. The focus groups script is detailed in Table 1.

**Table 1:** Semi-structured questions used to engage with the directors

Questions
1. In light of the presented data and the daily reality you are familiar with, do you consider a change in genetic healthcare necessary? In what direction? For what purpose?
2. How do you perceive the current state of genetic healthcare provision in Portugal compared to other European countries?
3. What institutions, structures, and entities would need to be part of a reorganization strategy?
4. What other specific actions do you suggest besides the strategies already identified with the Service Directors?
5. Which strategy would you prioritize as the most important?
6. Now that you have arrived at the top 3 priority strategies for change, how would you implement them? What specific actions would be involved?

As is customary in focus groups, the questions served as catalysts for discussion. To gather participants’ opinions, we employed various techniques tailored to the specific objectives of each question. For question number 2, we utilized an online survey with yes or no responses. For question number 3, we employed Mentimeter, an interactive online tool available on the Zoom platform, to create a word cloud. For question number 6, we used the same tool for participants to rank the strategies from most to least prioritized.

Considering the varied composition of the groups, which included service heads, junior professionals, and experienced professionals, we acknowledged that employing Mentimeter would enhance transparency and motivate participants to concentrate on subjects of personal significance. Responses were presented anonymously on the screen as participants contributed comments, acting as a catalyst for further discussion. The remaining three questions were deliberated verbally, sparking lively discussions, interactions, and dialogues among participants.

The focus groups took place in January, February, and April 2023 through the ZOOM platform.

The first focus group included five medical specialists, the second group had nine medical specialists, and the third group comprised five medical specialists. The three conducted focus groups lasted for 3 h and 9 min, with an average duration of 1 h and 3 min each. Finally, the group interviews were fully transcribed for analysis.

### Data Analysis

The transcribed audio recordings were separately analyzed by two of the authors (CC and LG). Subsequently, individual conceptual diagrams were crafted, emphasizing key ideas and emerging concepts. These individual analyses were then exchanged with a third researcher (MP), who conducted a comparative analysis. No predefined coding categories were established, and themes emerged from the data. Through collaborative discussions, common themes were identified and refined. In cases of discrepancies, the group engaged in discussions, with researchers presenting their perspectives. If differences persisted, the majority viewpoint was adopted.

Braun and Clarke’s thematic analysis approach (Braun and Clarke 2006) was employed to deepen understanding of the topic by identifying and analyzing patterns of meaning from the collected data. Given its comprehensive and flexible nature, a thematic analysis allows for identifying common themes to gain insight into participants’ perceptions, beliefs, attitudes, and experiences (Nowell et al. 2017).

Furthermore, a thematic analysis is suitable for studies with small samples, where depth of analysis is more crucial than statistical representativeness (Fugard and Potts 2015).

### Ethics

The Ethics and Responsible Conduct in Research Committee of the Institute of Research and Innovation in Health approved the study. Participants provided verbal informed consent to participate in the focus groups after receiving written and oral information about the study.

## Results

Following the methodological assumptions utilized in this article and the study’s objectives, four main conceptual themes emerged: (1) the influence of the European context in a national change; (2) Improvement of medical genetics education; (3) Transformation of the structure of medical genetics services; (4) Operationalizing change.

**Fig. 1:**
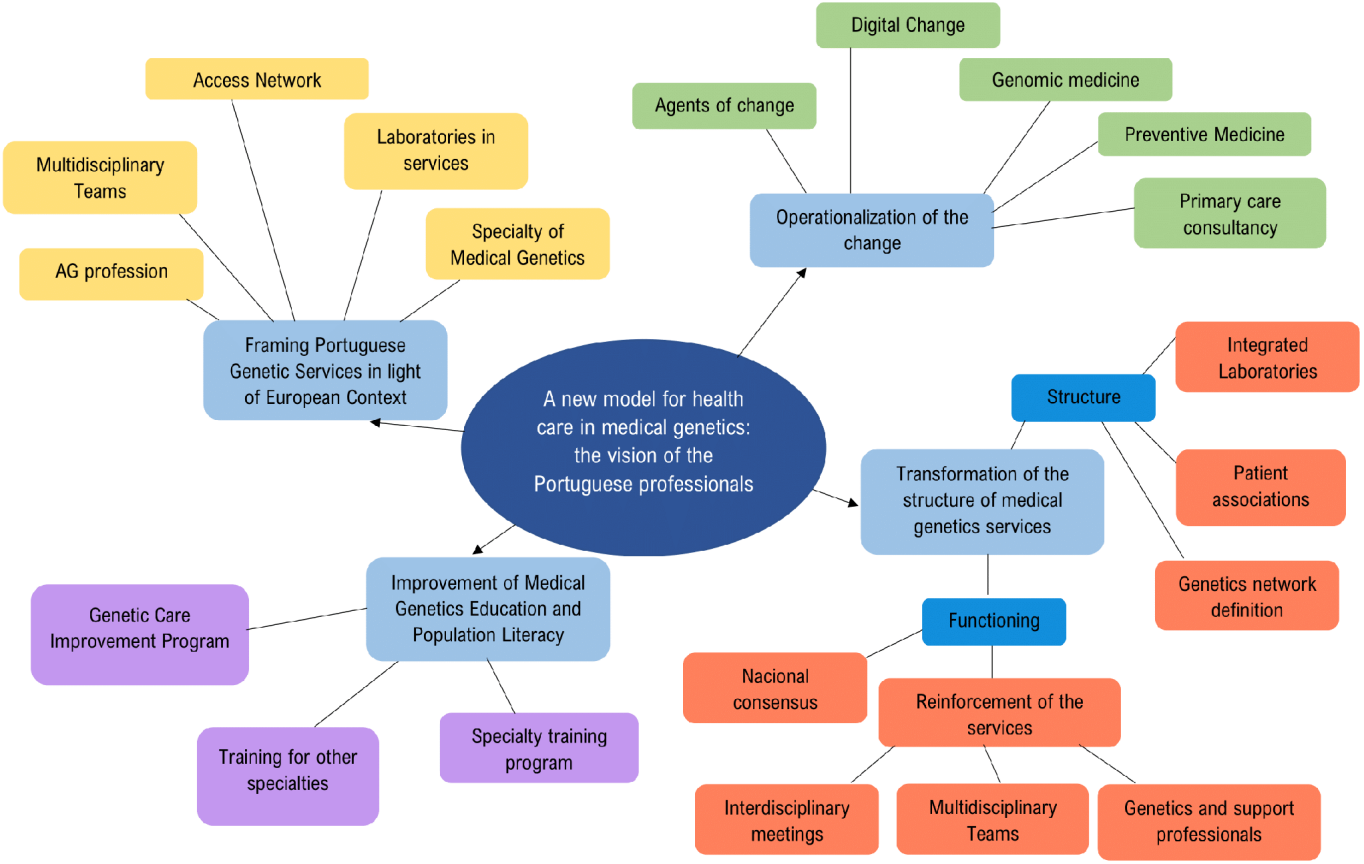
Dimensions and sub-dimensions resulting from the analysis of the results

Figure 1 illustrates the structure of these conceptual categories, including the subthemes.

The challenges faced by the medical genetics services emerged as a thematic block, which we do not consider relevant to explore as qualitative data since they have already been extensively investigated in a previous study by the researchers (Costa et al. 2023). However, we find it pertinent to mention for better contextualization of the conceptual themes we present next.

### Difficulties

Medical genetic specialists mentioned the following main challenges: excessive workload, leading to “voluntary” extra-hour efforts, scarcity of resources, particularly non-medical and logistical resources such as administrative staff, nurses, and psychologists.

Specialists also highlighted the need for training more medical geneticists and considered creating documentation in Portuguese as a pressing need for educating patients and other medical professionals.

Other difficulties pointed out were the lack of funding for services, bureaucracy, and the lack of quality indicators and routines for service improvement. Some highlighted problems included the waste of resources resulting from the lack of control over the patient’s journey and the absence of global guidelines from the National Health System, the new Executive Directorate, or the Specialty College of Medical Genetics.

### Theme 1: framing Portuguese genetic services in light of European context

Regarding the quality of Portuguese genetic services, specialists express no doubt that Portugal holds a favorable position, mainly due to the efforts of both medical and non-medical professionals. In various countries, there are heavy restrictions on access to services and genetic testing, which is not the case in Portugal.

(…) the experience we have, even in consultations, of receiving people from other countries here, often report difficulties in accessing consultations, in accessing the possibility of performing molecular tests - this doesn’t happen in Portugal (…) (P2).

On the other hand, unlike in Portugal, medical genetics is not a recognized specialty in other countries.“(…) I’m aware that there are still countries that aren’t as well off as us, especially those countries where the specialty isn’t even considered independent from other specialties. They are just doctors from other specialties with a differentiation, a sub-specialization in medical genetics. (P3)

The specialists also mentioned that our country provides quality healthcare to patients, and that referral is an aspect that has been worked on and has shown some results in recent years.

Portugal it’s certainly above average and median of European countries in terms of accessibility and quality of genetic care for residents in those countries. (P4)

According to the specialists, the factors that hinder a better position in medical genetics in Europe are the absence of multiprofessional teams and the lack of recognition of the genetic counselling profession and laboratories’ place integrated at services.

(…) sometimes, I receive reports from Portuguese families who underwent studies in other countries and … the technical and care aspects are very similar to what we do here, but on the side where they describe the service, they describe it with many professionals from various categories. When I see ours, it’s headed by doctors, a long list of doctors (…). (P6)

I think having laboratories integrated into the genetic service or at least having a close relationship with genetics or genomics laboratories and services… that proximity is what’s lacking the most. (P1)

### Theme 2: Improvement of Medical Genetics Education and Population literacy

Specialists also considered it essential to establish an enhancement program for genetic care, which should involve the ongoing update of Orphanet Portugal or the creation of a new free public portal where the most current information about rare diseases could be accessed.

(…) this issue of a free public portal with information is a very high priority. (…) (P7).

(…) a program for improving genetic care should include information for patients as well as for other colleagues (…) (P7).

The specialists emphasized the need for valuable and up-to-date documentation in Portuguese since the population’s health literacy level is very low.

(…) Health literacy in our population is very low (…) I think that the aspect of health literacy in Portugal and specifically in genetics is a strategy that I believe could potentially help. (…) (P11).

Another aspect pointed out was the medical genetics’ and other specialties training programs, through updating their educational curriculum, to develop skills and be better prepared for prescribing tests and referring.

(…) to reformulate the training program for the specialty itself and to suggest to other Colleges, other specialties, the reform of these programs in light of this. (P1)

### Theme 3: Transformation of the Structure of Medical Genetics Services

#### Structure

The participants considered a profound reorganization of the care and service structure necessary, redefining the medical genetics network, hospitals, and its services.

(…) there should also be some national guidelines on what network we should have (…) If we knew which services we need to develop, we’d know what vacancies to demand and what kind of structure we need in each service. (P6)

The change also calls for strengthening services by hiring more genetics professionals and other professional orders.

(…) we have to increasingly think about the overall structure of the service, not just in terms of the medical clinical staff, right? We urgently need to have other professional orders around us and work as a team (…) (P3).

Participants consider the recognition and integration of genetic counsellors as a crucial priority. Including genetic counsellors is regarded as a key requirement for achieving the training suitability of genetics services.

(…) I believe that genetic counsellors indeed have a role that could become very important within genetic services (…) and I think it can improve productivity, undoubtedly. (…) (P5).

The participants also mentioned the need for better integrating existing clinical genetics laboratories into the main genetics medical services network and establishing stronger connections with patient associations.

(…) it’s usually important to include clinical laboratory geneticists who help bridge the gap with genetics laboratories. Here, it’s not only genetic counselling professionals, but also clinical laboratory geneticists, administrative staff, and others that we don’t have. (P1)

(…) The easiest way, although difficult, is through patient associations. They have much more visibility than a small group of medical professionals or genetic counsellors (…) (P4).

#### Functioning

The change also calls for strengthening services by increasing the frequency of multidisciplinary meetings with other medical specialties and establishing multiprofessional teams with the inclusion of genetic counsellors, psychologists, social workers and clinical geneticist laboratory.

(…) I think maybe another item missing here is the promotion of creating multidisciplinary teams with all medical specialties (…) It has been done in recent times, and a lot has been invested in it, with great benefit. (P5).

Regarding the procedures for operationalizing change, the experts consider it essential to establish a national consensus to ensure good clinical practices and harmonize procedures performed by different services in the country.

(…) We will have to change the models because I think the waiting list is increasing in all services, we have numerous requests, and if we don’t change the models of operation here, it’s going to be very complicated in the future, right? (…) (P9).

(…) I think it’s important for us to establish more national consensus. I think in that aspect, we are a bit behind. We have, to some extent, the best practices from various services, but a national strategy is lacking for the sake of equity. (…) (P5).

### Theme 4: operationalizing the change

#### What to change?

**Fig. 2:**
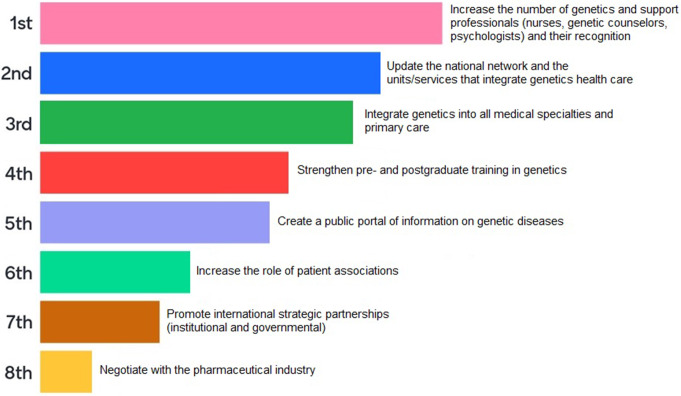
Order of priority strategies, according to the participants’ opinion

According to the opinion of experts present in the focus groups sessions, the most priority strategies (Fig. 2) are:

1. Increase the number of genetics, genetic counsellors, nurses and psychologists and their legal recognition in Portugal;
2. Update the national network and units/services that integrate health care in genetics;
3. Integrate genetics into all medical specialties and primary care.

#### Who should be involved in the change?

Relevant entities such as the Ministry of Health, the National Health Service Directorate, the Central Administration of the Health System (ACSS), the Groupings of Health Centers (ACES), hospital and local administrations, the Genetics Services themselves, and their clinical leadership were mentioned. Additionally, the enhancing role of other entities such as the Order of Physicians and the College of Medical Genetics Specialty, notably the Portuguese Society of Human Genetics (SPGH), diagnostic laboratories, and the Portuguese Association of Genetic Counselling Professionals (APPAGen), was referred.

#### What actions should be employed to execute the change?

The experts emphasize the importance of keeping up with digital change through the creation of an app, a tool for patient guidance throughout their healthcare journey (e.g., what resources are available, who are the specialists, which patient associations exist, informational material, etc.). This shift in services should also keep pace with the revolution in genomic medicine and, lastly, the need for a more preventive approach to healthcare services and the role of primary care through a closer relationship model with genetics.

A list of recommendations to improve the healthcare model in medical genetics (Table 2) was compiled, combining the participants’ suggestions in the various focus groups.

**Table 2:** Recommendations to improve the provision of medical genetics services

Recommendations
- Develop the integration of genetics in primary care and equip general practitioners with tools, training, and knowledge in clinical genetics.
- Ensure that all citizens have equal access to genetic services, regardless of geographic location. To achieve this, it is necessary to design a strategy to expand the reach of genetics services in more rural and remote areas.
- Implement educational programs for healthcare professionals and the general population, aiming to train professionals for patient care in genetics and promote health literacy. Online classes/courses and educational materials for distance learning for healthcare professionals can be created, focusing on empowering distant or overwhelmed centers.
- Improve coordination between genetics services and other medical specialties to ensure integrated and comprehensive care.
- Include all stakeholders in the change project: primary care professionals, medical geneticists, specialists from other areas, nurses, psychologists, genetic counsellors, molecular geneticists, healthcare institutions, patient associations, and local and political authorities.
- Promote a national network in genetics based on multidisciplinarity, multiprofessionalism, cohesion, sharing, and mutual support.
- Involve different social entities, national authorities, and local healthcare agents in the implementation strategy.
- Formulate, plan, and assess policies and strategies for continuously developing genetics and achieving excellence in these activities.

These recommendations can serve as a starting point for future initiatives and policies related to genetics and healthcare.

## Discussion

We opted for an inclusive approach to enable an in-depth exploration of the experiences and needs of medical genetics professionals and provide the opportunity to propose changes for genetic health services in Portugal. The co-produced recommendations highlight opportunities to enhance awareness and equity in accessing genetic health services.

The overall results of the conducted studies highlight the emergent need for significant transformations in Medical Genetics services in Portugal. The present findings underscore evidence of a shortage of human resources, the lack of an updated model for providing genetic care, and the necessity for more targeted and tailored public policies addressing the deficiencies in these services. All these fundamental challenges seem to require immediate attention to ensure efficient and high-quality patient care, considering the importance of maintaining services in the country at an internationally competitive level. Consistent with the available literature, there arises the need for a multidisciplinary and multiprofessional approach, investment in education and training, the integration of new professional roles, and the implementation of specific policies as crucial strategies to improve the quality and access to health services for those affected by genetic diseases in Portugal (Magalhães et al. 2016; Guimarães et al. 2024).According to the participating specialists, increasing the number of genetics professionals and other professional orders in medical genetics services is a priority strategy to address current challenges. The recommendation from the Royal College of Physicians UK for a ratio of 6–12 genetics professionals per million population underscores the importance of having an adequate workforce to meet the population’s needs (Abacan et al. 2018). However, few countries are close to meeting this recommendation, resulting in appointment delays, growing waiting lists, and burdening existing professionals (Dragojlovic et al. 2020; Maiese et al. 2019).

The stress resulting from workload overload, combined with the inherent responsibility of interpreting crucial genetic information for patients, can contribute to burnout and exhaustion (Hodkinson et al. 2023). This empirical work encountered a class of professionals exhausted, overwhelmed in the midst of several tasks, and struggling against ever-growing waiting lists for consultations. In this regard, the latest Annual Report on Access to Healthcare in SNS Establishments and Conventional Entities (Ministério da Saúde 2019) indicates that only 62.9% of the country’s Medical Genetics consultations occur within the guaranteed Maximum Response Time (TMRG). These data demonstrate the exceptionally challenging situation of Portuguese genetic services.

Hiring more genetic professionals, such as clinical geneticists, molecular geneticists, and other genetic specialists is one solution (Mikat-Stevens et al. 2015; Stoll et al. 2018; Costa et al. 2023) to meet the growing demand driven by technological advances in genomics and the increasing need to address a broader range of genetic conditions (Paneque et al. 2015).

Several other studies have evidenced the need to reinforce human resources at genetic services, as a crucial factor in enhancing the responsiveness of genetic services (Leach et al. 2017; Madlensky et al. 2017). Strengthening the teams helps reduce disparities in access to genetic services (Mikat-Stevens et al. 2015), allowing clinicians to focus on tasks aligned with their specific training (Stoll et al. 2018). These actions combined lead to more efficient and quality care delivery, improving patient outcomes.

The study participants also emphasize the importance of integrating a more collaborative and multidisciplinary healthcare service delivery model. The shift from a model centered around a single specialist to a collaborative model involving various professionals such as clinical geneticists, specialists from other medical fields, genetic counsellors, nurses, psychologists, and social workers is highlighted as an essential strategy to ensure the growth of Medical Genetics services and a holistic and more effective approach to the treatment of genetic conditions (Jamal et al. 2020; López-Fernández et al. 2020). This aligns with findings from other scientific studies, emphasizing the need for multiprofessional teams, with genetics experts working in collaboration with professionals from various fields, to provide comprehensive and personalized care (Gupta and Endrakanti 2023).

Furthermore, other medical genetics professionals, such as genetic counsellors and genetic nurses, play a significant role in providing comprehensive and quality care. Integrating genetic counsellors into medical genetics services already brings substantial benefits, as their skills complement those of genetic physicians (Paneque et al. 2017). The role of genetic counsellors could become of paramount importance in Portugal at the interface between Genetics services and the community, especially with patients and the associations that aim to represent them, playing a crucial role in promoting health in Genetics: providing greater accessibility, understanding, and emotional support for communities affected by genetic diseases (Roulston et al. 2023). Genetic counsellors offer psychosocial support, helping patients cope with the emotional impact of their condition, make informed decisions about genetic testing and treatments, and address ethical and moral issues (Lally e Laurino 2022). From another perspective, genetic counsellors play a vital role in raising awareness of genetic issues in the community (Lara-Otero et al. 2019; Ormond et al. 2023). They may be involved in educational programs, workshops, and awareness campaigns to inform the public about the importance of Genetics, prevention, and available services.

From a healthcare perspective, the participating professionals address the urgency of updating the Referral Network, which defines the main public genetics services and private institutions. Determining this network will optimize resources and ensure a comprehensive and collaborative approach to enable equal access to specialized genetic care.

The issue of the referral network is a comprehensive challenge that transcends the specialty of Genetics and affects various areas of healthcare in Portugal. The participating experts, while acknowledging the need for significant change, find themselves unable to lead effective proposals due to the workload that characterizes their services. Lack of time and resources to dedicate to restructuring initiatives becomes a significant barrier to promoting changes in the referral network. This situation reflects a broader dilemma in the healthcare system, where workload compromises the capacity of professionals beyond their direct clinical responsibilities. Finding solutions requires not only a desire for change but also a context that provides the necessary resources and support for Medical Genetics specialists and other healthcare professionals to collaborate in defining effective improvement proposals. The structural limitations in Medical Genetics services in Portugal itself reflect significant resource shortages and the urgency of defining this network. The lack of structure, evidenced even in the shortage of offices to accommodate different specialists, is an example of the severe physical limitations that services face.

This infrastructural deficiency not only compromises patient comfort but also impacts the efficiency and quality of services provided by professionals, limiting the ability of services to hire new professionals to expand care. To promote sustainable growth and meet the needs of the population, it is crucial to invest in expanding and modernizing the infrastructure of Medical Genetics services.

On the other hand, integrating reference centers and laboratories into this network will allow differentiated and highly specialized care provision. Moreover, these specialized centers are at the forefront of research on rare diseases as they have access to the latest scientific advancements and can rapidly implement new treatments and therapeutic approaches (Diário da República 2015). Portugal’s participation in European reference networks that bring together healthcare providers across Europe is also extremely important to facilitate the debate on rare diseases and concentrate available knowledge and resources.

The update of the Medical Genetics referral network dating back to 2004 (Direcção-Geral da Saúde, 2004), with the formation of a multidisciplinary working team, is thus fundamental and marks a significant step in addressing the present and future challenges involving genetic services in Portugal. The inclusion of representatives from the Directorate-General of Health, various genetics services in the country, and members of the Specialty College, as suggested by participating experts, will establish a collaborative approach that integrates different perspectives and experiences. The participation and leadership of experts from other areas of healthcare, familiar with the context of Medical Genetics, are crucial to ensuring a comprehensive and informed view and the timely definition of the current structure of genetic care. The role of this working team goes beyond a simple update of the network; it has the potential to redefine strategies, strengthen coordination, and promote efficiency in Medical Genetics care in Portugal.

The participating specialists also highlight the importance of the role of the genetic physician in care coordination and communication with other healthcare professionals. They emphasize the need for training and educating other healthcare professionals in genetics, encouraging collaboration, direct supervision, and interdisciplinary meetings as described in other countries (Unim et al. 2017, 2020; Tizzano Ferrari 2017; Cassiman 2010; Roberts et al. 2014).

Participants emphasized the importance of policies focused on undergraduate and postgraduate training in medical genetics as a fundamental means to improve the knowledge and competence of healthcare professionals from other specialties. Gaps in genetics knowledge can lead to significant medical errors with medical, ethical, financial, and psychosocial implications for patients and their families, such as requesting the wrong test, misinterpreting test results, or incorrectly assuming a variant (Farmer et al. 2019, 2021). In this regard, efforts should ensure that all medical and multiprofessional specialties have a general knowledge of medical to promptly refer patients to specialized centers whenever possible (Strnadová et al. 2022).

In this context, particularly General Practitioners (GPs) are crucial in filtering information and guiding patients faced with the growing demand for genetic tests, ensuring they understand the implications, limitations, and potential risks (Magalhães et al. 2016; Paneque et al. 2016). Strengthening genetics training for GPs and providing services for Medical Genetics consultation can be effective strategies to bridge the gap between relatively low demand and the need for specialized knowledge in Genetics, ensuring that all patients have access to accurate information and appropriate guidance when needed (Harding et al. 2019; Cusack et al. 2021).

To assist in this primary healthcare consultation process, genetic counsellors can play a relevant role as guides, assisting GPs in scheduling appointments, interpreting genetic test results, and facilitating communication between patients and Medical Genetics services (Carroll et al. 2019; Slomp et al. 2022).

In following this shift, digital health programs can emerge as an innovative solution to overcome geographical barriers and facilitate access to genetic care for a broader range of patients. The implementation of teleconsultations and professional counseling through digital platforms provides an opportunity to overcome physical limitations, allowing individuals in remote areas or with reduced mobility to access specialized genetic services without the need for extensive travel (Dantas et al. 2023).

Furthermore, the integration of digital genetic counselling can play a crucial role. By offering remote guidance, these programs can facilitate efficient communication among healthcare professionals, enabling General Practitioners to obtain specialized advice from genetics professionals for specific cases. This not only optimizes the efficiency of care provision but also promotes a collaborative and interdisciplinary approach to managing genetic issues (Bombard et al. 2022; Cazzaniga et al. 2022). However, it is essential to ensure that such programs maintain high standards of security and privacy, along with the inclusion of ethical guidelines to ensure quality and responsibility in the provision of these digital services in the field of Medical Genetics.

Adopting standards of practice in genetic healthcare is another essential aspect pointed out by participants, who advocate for the existence of more effective models, national guidelines for pathologies, and national consensuses on genetic counselling, etiological investigation, etc. Despite adhering to the norms of the European Board of Medical Genetics (EBMG), experts believe that national standards can help harmonize differences between genetic services regarding education and genetic practice,, ensure that professionals are aligned with best practices and updated guidelines, and regulate the quality and competence in the provision of genetic services (Paneque et al. 2016). This regulation contributes to patient safety, result reliability, and the assurance of ethical and responsible practice (Unim et al. 2019).

In the current context, Portugal continues to work on improving genetic services to meet the needs of the Portuguese population. The practice of Medical Genetics in Portugal reveals a challenging scenario, as the growing demand for these services is not aligned with the current capacity of the health system. Understanding this local context is essential to assess the relevance of studies and research related to medical genetics in Portugal and better inform genetic health practices and policies.

The implementation of a change in Medical Genetics services in Portugal is therefore an urgent necessity, considering the significant transformations brought about by genomics in healthcare services worldwide. However, it is essential to maintain a realistic view of all these issues and know where to start: structural and concerted changes are not possible without national political interventions.

Perhaps the first step is even prior to the conclusions raised in this study, involving a global psychoeducation about genetic diseases that, more than being rare, have a significant impact on those living with them in their identity. Geneticists can already begin to make some small changes in their genetic services (e.g., improving coordination with other specialties, promoting networking with other geneticists). Later, with better working conditions and more professionals in the services, educational programs can be implemented, and a public portal/mobile application with genetic information can be created. At a more challenging higher level, political involvement will be necessary for implementing changes in primary healthcare and recognizing the profession of genetic counsellor.

Only effective coordination among all these instances and bodies responsible for health, education, and social intervention policies can make the future directions presented here a reality.

### Limitations

Using a convenience sample may restrict the representativeness of professionals from genetic services. However, all public medical genetic services were included in the study.

The lack of a publicly available and up-to-date listing of healthcare entities offering genetic consultations and their professionals hindered the sampling process and may have influenced the results. Nevertheless, it is important to emphasize that the study is exploratory and reflects the specific reality of hospital genetics in Portugal. Caution should be exercised in interpreting the results. However, the study is relevant as it provides crucial insights into the current state and needs of genetic services, highlighting the imminent need for structural change.

## Conclusion

The findings discussed herein highlight a stark misalignment between the escalating demand for genetic services in Portugal and the constrained capacity of the National Health Service (SNS) to meet this burgeoning need. Urgent action is imperative to bring about a transformation in Medical Genetics services in Portugal, considering the profound shifts brought about by genomics in healthcare services globally.

Continuous education, genetics training, and updating guidelines for best practices are essential to ensure the quality of healthcare in medical genetics. Implementing strategies that promote education and health literacy is fundamental for having healthcare professionals who are up-to-date and equipped to deal with advances in genetics and provide quality services to patients.

A new care model with a multidisciplinary, multiprofessional and collaborative approach within healthcare teams is necessary in Portugal. Therefore, the integration of professionals from different specialties is required to remove barriers and ensure that individuals with genetic diseases receive the necessary care. Translating evidence from international research into clinical practices is of utmost importance to ensure that advancements in genetics are applied meaningfully for the benefit of patients. This translation requires a commitment to adopting evidence-based practices and a continuous improvement mindset.

For all this, the results of this study are intended to have contributed to informing urgent decisions surrounding the implementation of structural changes in Portuguese genetics services.

## Data Availability

The authors declare they followed the protocols of their work center on data publication.
